# Comparison of self-etching and conventional bifunctional coupling agent in lithium disilicate ceramic: A systematic review and meta-analysis

**DOI:** 10.4317/jced.61369

**Published:** 2024-03-01

**Authors:** Juliana-Lujan Brunetto, Aldiéris-Alves Pesqueira, João-Paulo do Vale-Souza, Lucas-Tavares Piacenza, Daniela-Micheline dos Santos, Marcelo-Coelho Goiato

**Affiliations:** 1 DDS,MSc, PhD student at the Department of Dental Materials and Prosthodontics, Araçatuba Dental School, São Paulo State University, Araçatuba, São Paulo, Brazil; 2 Profesor at the Department of Dental Materials and Prosthodontics, Araçatuba Dental School, São Paulo State University, Araçatuba, São Paulo, Brazil; 3 DDS,MSc, PhD student at the Department of Dental Materials and Prosthodontics, Araçatuba Dental School, São Paulo State University, Araçatuba, São Paulo, Brazil; 4 DDS,MSc, PhD student at the Department of Dental Materials and Prosthodontics, Araçatuba Dental School, São Paulo State University, Araçatuba, São Paulo, Brazil; 5 Profesor at the Department of Dental Materials and Prosthodontics, Araçatuba Dental School, São Paulo State University, Araçatuba, São Paulo, Brazil; 6 Associate Profesor at the Department of Dental Materials and Prosthodontics, Araçatuba Dental School, São Paulo State University, Araçatuba, São Paulo, Brazil

## Abstract

**Background:**

The aim of this systematic review is to determine the effectiveness of self-etching primers in comparison to the conventional protocol with hydrofluoric acid and silane treatment for bonding lithium disilicate ceramics.

**Material and Methods:**

The formulated PICO question for this research was: “Does self-etching silane primer surface treatment in lithium disilicate ceramics present a similar bond strength value compared to conventional hydrofluoric acid and silane treatment?”. Combinations of words and appropriate truncations were adapted for each database. For the selection, duplicate articles were systematically eliminated using Mendeley software. The Cohen’s Kappa statistic was then computed, RoBDEMAT questions were addressed, and the meta-analyses were conducted using RevMan 5.4, at a significance level of 5%.

**Results:**

Two independent reviewers conducted a blind and independent analysis of 190219 articles from PubMed, Scopus, Web of Science, and OpenGrey. Subsequently, they extracted data from 21 studies for the systematic review and in 16 the meta-analysis. In all in vitro studies, the most frequently cited concentration of hydrofluoric acid was 5%. In the meta-analysis, no statistical differences were observed between the two treatments concerning bond strength.

**Conclusions:**

Self-etching silane primers demonstrate promising results in lithium disilicate bonding, suggesting their potential as an alternative surface treatment to hydrofluoric acids + silane.

** Key words:**Lithium disilicate, Hydrofluoric acid, Dental Porcelain, Ceramics, Silanes.

## Introduction

Even though lithium disilicate injection was patented in 2002, it remains one of the most commonly used and studied materials (1). In comparison to other glass-ceramics, lithium disilicate stands out as an aesthetically pleasing and relatively resilient material. Comprising 70% lithium disilicate crystals and a 30% vitreous matrix, it incorporates inorganic particles of silanized barium and colloidal silica, thereby facilitating chemical adhesion.

Glass-ceramics can achieve chemical cohesion with the inorganic components, such as silica, present in resin cement, specifically through the chemical union with Bis-GMA (bisphenol A-glycidyl methacrylate) and TEGMA (triethylene glycol monomethacrylate) (2). And these steps, with a bifunctional coupling agent (silane), are essential for achieving both mechanical and chemical microretention (3).

Different methods have been employed in clinical practice to establish a reliable and enduring bond, and hydrofluoric acid (5-10%) has traditionally served as the gold standard for surface conditioning (4,5). However, in addition to the dental surface conditioning step, the removal of the outermost layer of silane is ideally desired, leaving only the most sTable and non-oxidized portion (6). Nevertheless, introducing additional clinical steps to a material (hydrofluoric acid) that already had potential toxicity due to pH levels, instability, and reactivity against oral cavity soft tissues, transforms it into a technique that is increasingly prone to errors and complicates the clinical procedures (4,5).

To overcome these limitations, novel self-etching silanes have been developed as an alternative, aiming to mitigate potential risks in the oral cavity and reduce technical sensitivity during clinical steps, albeit with some controversy. Simplifying the bonding procedure into a single step requires the product to chemically unite incompatible solutions, which may compromise its adhesive strength (6). Therefore, this systematic review aimed to analyse the effectiveness of lithium disilicate ceramic coupling agent provided by the application of self-adhesive silane primer compared to the hydrofluoric acid conditioning protocol followed by the application of conventional silane.

## Material and Methods

-Registration and standardization study protocol

This systematic review followed the guidelines of the Preferred Reporting Items for Systematic Reviews and Meta-analysis (PRISMA) (7) and was registered at the International prospective register of systematic reviews - PROSPERO (CRD42021252016).

The studies were included based on the PICO question: “Does self-etching silane primer surface treatment in lithium disilicate ceramics present a similar bond strength value compared to conventional hydrofluoric acid and silane treatment?” determined by: Participant (P)-Lithium disilicate ceramic; Intervention (I)-Self-etching silane primer surface treatment; Comparison (C)-Conventional hydrofluoric acid and silane surface treatment; Outcome (O)-Bond strength analysis.

-Eligibility criteria

The applied search strategy inclusion criteria for selecting studies were: 1) *in vitro* studies; 2) with bond strength (MPa) analysis by shear, microshear, tensile, or microtensile tests; 3) lithium disilicate ceramic surface; 4) self-etching silane primer and hydrofluoric acid treatment with silane treatment groups; and 5) published until 07/27/2023. Exclusion criteria were studies that: 1) incorporated additional types of surface treatment; 2) were duplicates or with different topics; 3) exclusively utilized self-etching silane or hydrofluoric acid; 4) were literature reviews, conference abstracts, or letters to the editor.

-Database selected and search strategy

The Medical Subject Headings (Mesh) keywords were combined with pre-determined Boolean operators with the asterisk to increase the search accuracy: “self-etching primer”, “single-step self-etching primer”, “ceramic primer”, “monobond etch and prime”, “hydrofluoric acid”, “coupling agent”, “silane”, “monobond”, “lithium disilicate”, “glass ceramic”, in 3 electronic databases (Medline/PubMed, Scopus, and Web of Science) and a manual search in Open Grey following the format from each database.

Additionally, articles were manually searched in the list of references of the included studies and when it was not possible to download the article, it was requested through the Bibliographic Switching Program (COMUT).

-Screening and selection of the papers

The studies included were independently evaluated by three investigators (J.L.B., J.P.V.S., and L.T.P.) and imported into Mendeley software (version 1.16.1) for duplicate removal. After, the data were organized using Microsoft Excel Professional Plus, the Cohen’s Kappa statistic was computed, and the risk of bias RoBDEMAT questions were answered and judged (8-11).

-Statistical analysis

The statistical analysis of the included studies was performed by employing a random effects model using Review Manager software (version 5.4; Cochrane Collaboration), at a significance level of 5%. The standardized mean bond strength values data were extracted to be analyzed in the meta-analyses, and the heterogeneity among studies was assessed via the Cochran Q test with a threshold *p-value* of 0.1 and by applying the inconsistency index (I2).

Furthermore, qualitative results were shown in Tables, and mean surface treatment values were performed in graphs for visual comparison.

## Results

**Figure 1:**
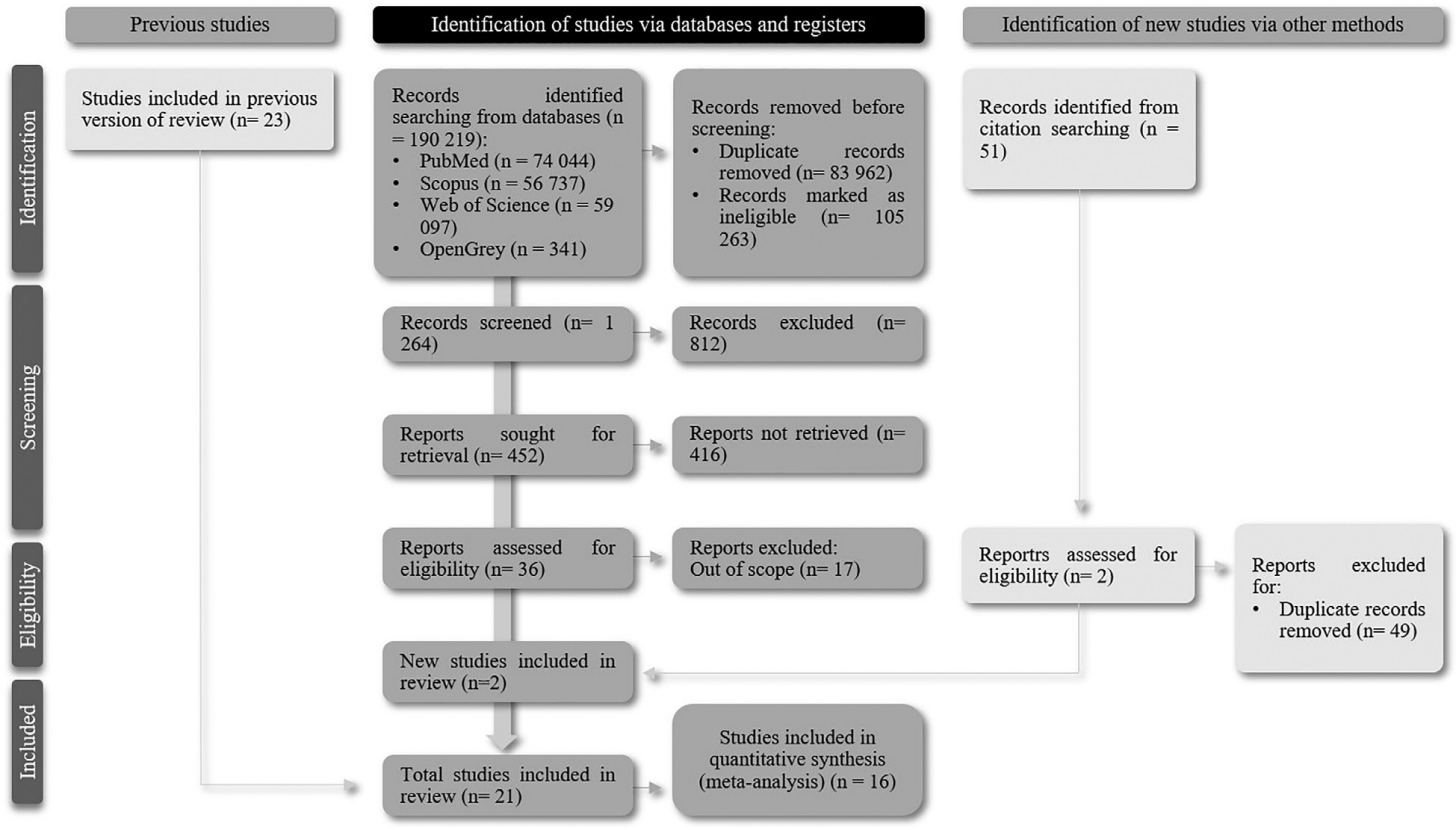
Flow chart of study selection according to PRISMA workflow.

To achieve greater precision in the database search, a preliminary investigation was performed. For the official search, a total of 190 219 studies were initially observed as presented in Figure 1, and after removing duplicate articles and studies that have a different topic than the proposed one, we reached 19 included articles. The references of previously selected articles were also analyzed, adding 2 other studies (searched in references) to the total of 21 studies in the qualitative and 16 meta-analyses/quantitative data, (Fig. 1).

Even without language and initial date search restrictions, all articles selected were in English and from 2017 to 2023. And, according to the Cohen’s Kappa (k) inter-rater statistical agreement, results were: “perfect agreement” for Open Grey (k = 1.00), “almost perfect agreement” for Web of Science (k = 0.92) and PubMed/MEDLINE (k = 0.81), and “substantial agreement” for Scopus (k = 0.76) (8).

To provide methodological clarity and robustness, a scheme of RoBDEMAT risk of bias categorization was used (Table 1,  Table 1 cont.): >70% low risk of bias (green); <70% medium risk of bias (yellow); and <50% high risk of bias (red) (8-11).

**Table 1:**
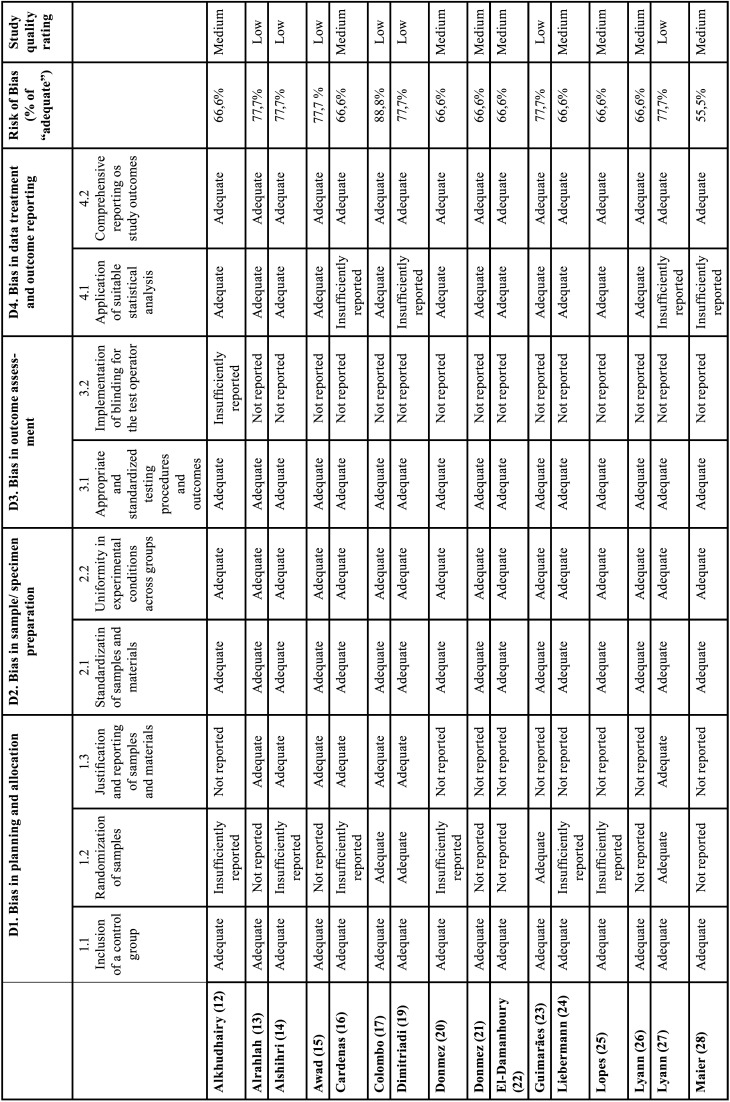
Review of In vitro studies quality rating according to the RoBDEMAT tool. Studies were classified as: >70% low risk of bias (green), <70% medium risk of bias (yellow) or <50% high risk of bias (red) (10,11).

**Table 1 cont.**
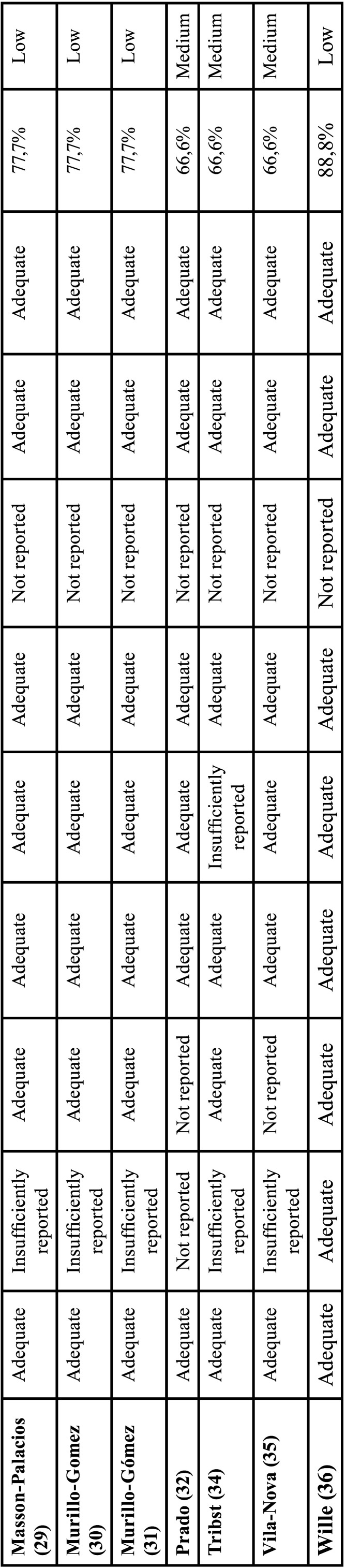
Review of In vitro studies quality rating according to the RoBDEMAT tool. Studies were classified as: >70% low risk of bias (green), <70% medium risk of bias (yellow) or <50% high risk of bias (red) (10,11).

These signaling questions were individually answered in Table 1 with most of the studies not reporting properly the “Randomization of samples” (D1.2) and the “Implementation of blinding for the test operator” (D3.2), nonetheless without “high risk of bias”.

While most of the authors report the results only of the lithium disilicate (12,14,18-19,22,24-26) or make comparisons with one more ceramic (feldspathic (16,21,23,27,30-32), zirconia-reinforced lithium silicate (15,20) and zirconia (33), Donmez *et al*. (20) (lithium disilicate, hybrid, zirconia-reinforced lithium silicate and, leucite-based) and Vila-Nova *et al*. (32) (lithium disilicate, hybrid, feldspathic and, resin-modified) were the studies that compared the greater amount of ceramic variety. Another comparison was made regarding the concentrations and action time of the hydrofluoric acids (20,29), lasers (12,19), or the 50 µm sandblasting particles (17,33) after performing the polishing protocol. Additionally, various concentrations of hydrofluoric acids (4.7% (15); 4.8% (21); 4.9% (18); 5% (13-14,16-17,19-20,24-29,33); 9% (23-24); 9.5% (20,24); 9.6% (24,12); 10% (17,22,24,29,31) were mentioned, with 5% hydrofluoric acid being the most commonly used among them (Table 2,  Table 2 cont.).

**Table 2:**
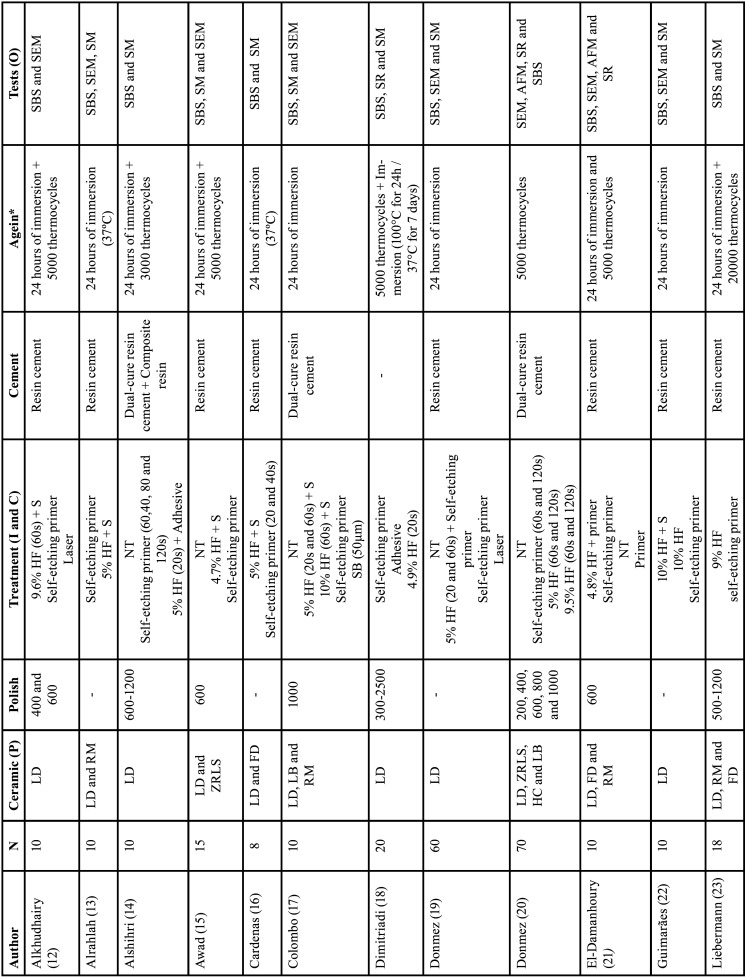
Qualitative data referring to the studies added to the systematic review.

**Table 2 cont.**
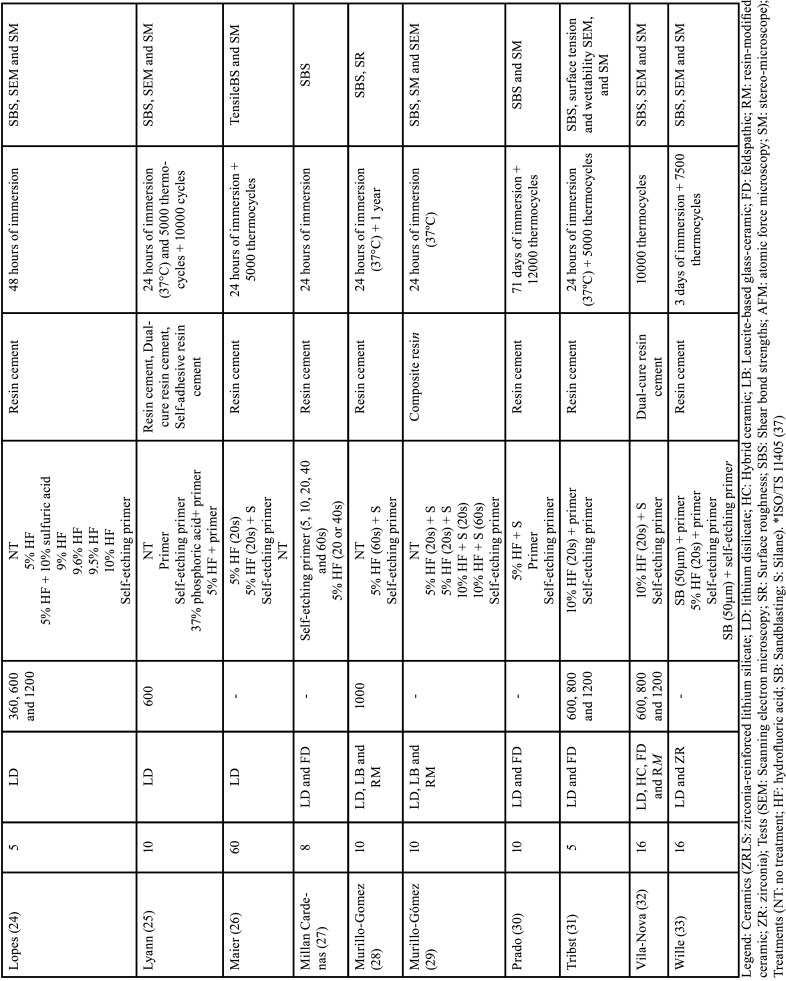
Qualitative data referring to the studies added to the systematic review.

Despite the variation in acids, it did not occur with other materials. Some authors did not provide a detailed description of their finishing and polishing protocol (13,16,19,22,26-27,29-30,33) or specify which type of cement18 was used, making the reproduction and comparison difficult.

It is important to note that all articles in this study adhere to ISO/TS 11405:2015 standards (34) for the bond strength testing.

As indicated in Table 3, all articles analyzed their samples during an ‘initial’ period after 24 hours of immersion in 37°C water and some proceeded with variations in thermocycling aging time (3.000 cycles (14), 5.000 cycles (12,15,18,21,25,26,31), 10.000 cycles (25), 12.000 cycles (30), or 20.000 cycles (23) at 5-55°C), extending the immersion period to 48 hours (24), or even 1 year (28) (Table 2, 2 cont.). But none of the mentioned experiments involved substances capable of inducing degradation or modifying the pH or chemical composition of the samples during immersions.

**Table 3:**
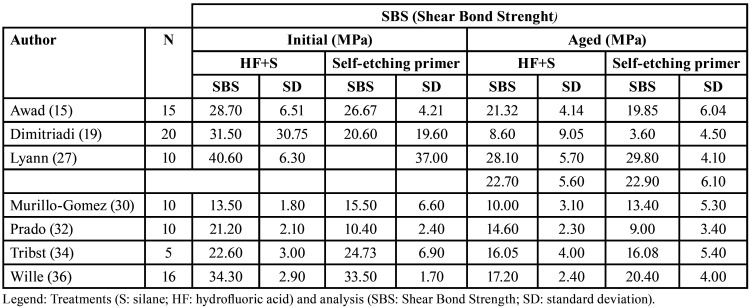
Quantitative data regarding the Shear Bond Strenght force of the selected studies.

**Figure 2:**
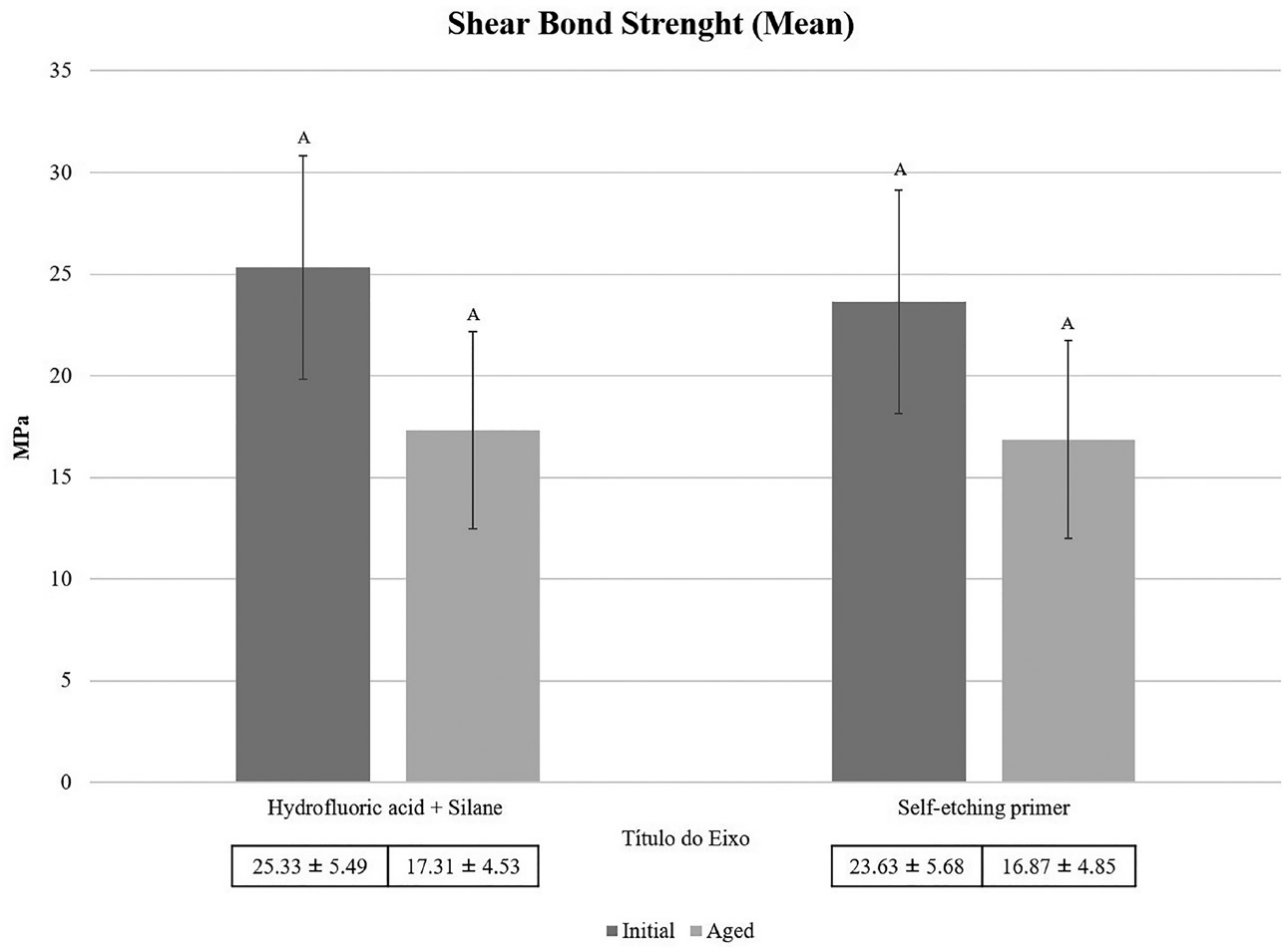
Shear bond strenght mean force graphic.

So, Figure 2 illustrates the shear bond mean force values, leading to the conclusion of no statistically significant mean difference between treatments.

Another point of comparison is the number of samples, Donmez *et al*. (19) presented the highest quantity (n=60), while, as expected, most articles exhibit n=1012-(14,17,21-22,25,28-30). Contrastingly, Tribst *et al*. (31) and Lopes *et al*. (24) presented a reduced number of only 5 samples.

-Meta-Analyses

**Figure 3:**
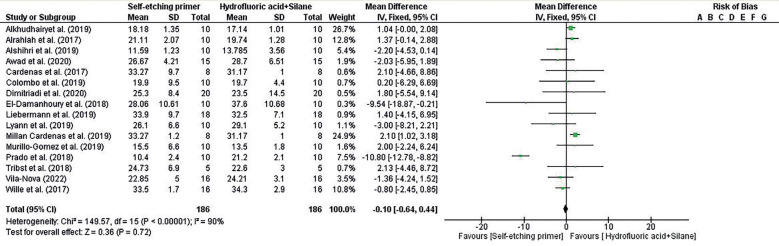
Forest plot of the initial comparison of shear bond strength with Self-etching silane primer and Hydrofluoric acid + Silane.

**Figure 4:**
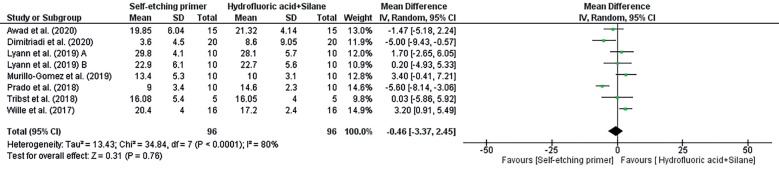
Forest plot of after aging comparison of shear bond strength with Self-etch silane primer and Hydrofluoric acid + Silane.

In the results of Figure 3 and Figure 4 the forest plot’s prism seems to lean more towards the “Self-etch silane primer” side but intersects the middle of the line, suggesting no statistical difference between the two treatments (Self-etch silane primer and Hydrofluoric acid + Silane) analyzed by meta-analysis. In the same Figure, the percentage of variation at I² indicates substantial heterogeneity between studies. The overall value for “Mean Difference IV” reveals a difference of -0.10 between the compared groups, representing their total effect across all studies.

## Discussion

This comprehensive approach ensures precise and reliable results in the analysis of bonding materials using resin cements. As indicated by the vast majority of the included studies, there is no discernible difference between the treatments of Self-etch silane primer and Hydrofluoric acid + Silane.

A limitation of this study is that the articles investigating the bonding of materials employed a variety of test types, including microtraction, macrotraction, microchecking, pull-out, push-out, and micro-push-out. And despite the abundance of published articles, the lack of standardization in tests, analysis periods, and the variety of ceramics remains an obstacle to their comparison.

In general, acids create effective micromechanical and chemical retention by removing the crystalline phase of silica-based ceramics (vitreous) (12). Despite the fact that 5% concentrations create sufficient porosities and irregularities, hydrofluoric acid with a 10% concentration is established in the literature (27).

Although reducing the concentration of hydrofluoric acid does not eliminate the symptoms caused by direct exposure (such as intoxication, dermal burns, eye lesions, acute gastrointestinal issues, respiratory problems, irritation and nasal inflammation, and bleeding of the mucosa) (5,35), the extent of tissue damage and toxicity depends on the acid concentration, the contaminated area, the age of the person, and the duration of exposure (35). And considering the clinical procedure and contact time, Millan Cardenas (27) analyzed different durations of active and passive application of Monobond Etch and Prime.

According to the manufacturer’s instructions, the active application should be done for 60 seconds to eliminate water, alcohol, and other silane products (27). This process helps increase the adhesions by completing the condensation reaction between silane and silica and promoting the formation of siloxane (28). When water is attached to the bases of the activated silane, it performs a crucial role in generating free silanol groups by dehydrating them and converting the solvents into oligomers (28,36), according to the temperature.

Wille *et al*. (33) also emphasize the importance of silanization before oligomerization and after hydrolysis36. Along with other studies (22,27-28,32) corroborate with this review, by observing no significant difference between the treatments, even after 12000 thermal aging cycles (30).

Despite that, the *in vitro* tests do not fully capture the complexity of aging that occurs in the mouth. And factors such as masticatory forces, temperature changes, and exposure to saliva, food, drinks, and oral microbiota can all impact the longevity of these restorations. As a result, it is important to consider these variables when assessing the effectiveness of ceramic restorations in the oral environment.

Based on the findings of this systematic review, it is hypothesized that the self-etching silane primer method can generate microtopographic surface changes comparable to those induced by hydrofluoric acid + silane treatment, thereby resulting in a similar coupling force. However, this study has inherent limitations, emphasizing the necessity for additional long-term and standardized analysis of ceramic restorations. And considering the positive results and limited clinical relevance of shear bond strength studies included in this review, it is concluded that the use of self-etching silane primer provides similar values to conventional hydrofluoric acid + silane protocol (HF+S), promoting an adequate coupling between cement and ceramic.
